# Expression and functional analysis of the Follistatin‐like 3 (FSTL3) gene in the sheep ovary during the oestrous cycle

**DOI:** 10.1111/rda.13879

**Published:** 2020-12-22

**Authors:** Jianning He, Qiuyue Liu, Shunyu Yu, Mengyuan Lei, Jifeng Liu, Ran Di, Zhaojia Ge, Wenping Hu, Xiangyu Wang, Nan Liu, Mingxing Chu

**Affiliations:** ^1^ College of Animal Science and Technology Qingdao Agricultural University Qingdao China; ^2^ Key Laboratory of Animal Genetics, Breeding and Reproduction of Ministry of Agriculture Institute of Animal Science Chinese Academy of Agricultural Sciences Beijing China

**Keywords:** expression, FSTL3 gene, function, seasonal breeding, sheep

## Abstract

Follistatin‐like 3 (FSTL3) is a regulator of cellular apoptosis and was previously identified via RNA‐Seq to be associated with follicular development in mammalian ovaries. However, the mechanism underlying the FSTL3 regulation of oestrus in sheep remained poorly understood. In this study, the oestrogen (E2) and progesterone (P4) concentrations in blood were detected, and the expression level and functional analysis of FSTL3 in the ovary were studied during the different reproductive stage in Aohan fine wool sheep (seasonal breeding breed in China). The concentrations of E2 and P4 at the anestrus were significantly lower compared to dioestrus, proestrus and oestrus stages. Higher expression levels of FSTL3 were observed in the sheep ovary, hypothalamus, and thyroid. During different reproductive stages, higher expression levels were found during the stages of dioestrus and proestrus, while lower levels were found during the oestrus and anestrus stages. Functional analysis of FSTL3 was performed in primary granulosa cells (GCs) of sheep. The concentration of E2 increased significantly after RNAi interference of FSTL3, while the P4 level decreased. FSTL3 can decrease P4 levels, which might be involved in mediating oestrous cycle in sheep.

## 1 INTRODUCTION

Reproduction is an essential function for animals that requires the coordinated action of both endocrine hormones and locally derived growth factors (Yoshimura, 2010). In mammals and other species, these regulatory factors mainly originate from the hypothalamic–pituitary–gonadal (HPG) axis. The ovary is a downstream gonadal tissue that can secrete E2, and P4. In response, E2 secreted by granulosa cells (GCs) triggers a series of physiological changes in ovaries, reproductive tract, vulva and behaviour (Bartness et al., 1993; Chemineau et al., 2010; Nishiwaki‐Ohkawa & Yoshimura, 2016). Normal levels of E2 and P4 secreted from GCs are essential for follicular growth and atresia (Bertoldo et al., 2012; Matsuda et al., 2012).

Most wild and domesticated species display seasonality in reproduction, which is controlled by photoperiod. Furthermore, the different responses of the hypothalamus to E2 according to annual photoperiodic cycle causes animal seasonal reproduction (Goodman et al., 1982; Karsch et al., 1993; Maeda et al., 2010). The sheep is a seasonal breeding species (Ortavant et al., 1988). Aohan fine wool sheep is a seasonal breeding breed in China.

Follistatin‐like 3 (FSTL3), originally called follistatin‐related gene (FLRG) (Hayette et al., 1998), and also known as follistatin‐related protein (FSRP) (Schneyer et al., 2001), is a type of secretory glycoprotein which belongs to the follistatin family. It is mainly expressed in the ovary, testis, placenta, embryonic trophoblast and endometrium (Kralisch et al., 2017; Oldknow et al., 2013; Robertson et al., 2013; Shi et al., 2011). Previous studies showed that transgenic overexpression of the FSTL3 gene in male and female mice caused sub‐fertility, and their gonads were significantly smaller compared to those of wild‐type littermates (Rebourcet et al., 2017; Xia et al., 2004). Deletion of FSTL3 not only increased testicular size but also prevented age‐related testicular regression in mice (Oldknow et al., 2013).These data suggest an important role of FSTL3 in animal reproduction. It was reported that FSTL3 mRNA abundantly expressed in the placenta, ovary, uterus and testis. Expression levels of FSTL3 in the placenta can continuously increase during the second half of rat pregnancy while levels of ovarian FLRG mRNA did not change during the rat oestrous cycle. Concurrency of FSTL3 expression with dioestrus and proestrus and the presence of CL (Arai et al., 2003), it was shown that the expression level of FSTL3 in the sheep ovary was higher at dioestrus and proestrus stages than expression levels at oestrus and anestrus stages from RNA‐sequencing results (Di et al., 2014). Previous studies showed that the expression level of FSTL3 was fluctuating in corpus luteal cells and GCs, and the higher levels were found at the middle stage of follicular development and the early stage of ovulation (Sun et al., 2019). FSTL3 expression decreased dramatically during ovulation and was almost undetectable after ovulation (Singh et al., 2019). The expression level of FSTL3 did not increase during the dioestrus stage until luteal cells emerged (Liu et al., 2002; Shi et al., 2011).

Our study was trying to characterize FSTL3 gene in mediating oestrous cycle alternates in sheep. The objective was to understand the mechanism underlying the FSTL3 regulation of oestrus in sheep. Functional analysis of the FSTL3 gene was conducted in primary sheep GCs using the RNA interference (RNAi) technique which can affect E2 and P4 levels in sheep GCs. FSTL3 was found to be a candidate gene that might mediate oestrous cycle regulation in sheep.

## 2 MATERIAL & METHODS

### 2.1 Animals and sample collection

All procedures involving animals were approved by the Animal Care and Use Committee of Qingdao Agricultural University, Qingdao, China and the Animal Care and Use Committee of Aohan fine wool (AFW) Stud Farm, Aohan, Chifeng, China.

AFW sheep were selected from AFW Stud Farm and housed in the same farm in Inner Mongolia, China. The twenty, 3‐year‐old, clinically normal and non‐pregnant ewes were examined daily for oestrous activity with a teaser ram from early summer to late autumn. The date and duration of oestrus were recorded, and blood samples were collected for measurement of serum hormone E2 and P4 concentrations. The oestrus was determined depending on obvious oestrous signs in response to the teaser ram. Anestrus is a state without obvious oestrous signs for 30 consecutive days (more than a whole oestrous cycle). Ewes in luteal phase and proestrus were determined according to records of three consecutive oestrous cycles, the dioestrus stage is five days before estrous, the proestrus stage is one day before oestrous (Goodman, 1994). Finally, twelve ewes were selected randomly, killed at the four different reproductive stages and three ewes in each stage. The whole ovaries were collected and immediately snap‐frozen in liquid nitrogen for total RNA extraction. E2 (B05TFB, Beijing North Institute of Biotechnology Co., Ltd.) and P4 (B08JFB, Beijing North Institute of Biotechnology Co., Ltd.) levels were measured via radioimmunoassay according to the instruction (Campbell et al., 1994; Djahanbakhch et al., 1981).

### 2.2 Cell culture and immunofluorescence staining

Fresh ovaries were selected from healthy adult female AFW sheep, added to phosphate‐buffered saline (PBS) at 4°C, and quickly taken back to the laboratory. After they were washed with 75% ethanol twice (about 10 s each), sterile saline was used to remove the alcohol. The other tissues were carefully removed. The visible follicles (> 3.0 mm in diameter) were selected for follicular fluid samples containing GCs. The GCs were separated from the follicular fluid via centrifugation for 5 min at 1,000 rpm and then washed with sterile Dulbecco's Modified Eagle's Medium (DMEM; high glucose, HyClone, Logan, UT, USA) twice. The cells were cultured in medium supplemented with 10% foetal bovine serum (FBS; Gibco, Waltham, MA, USA) and 1% penicillin‐streptomycin solution (Gibco) and then incubated at 37°C under 5% CO_2_ in humidified air.

Immunofluorescence staining was used to identify the follicle stimulating hormone receptor (FSHR), which is specifically expressed in granulosa cells. Cells were fixed with 4% polyoxymethylene and then washed with PBS; 10% Goat Serum in 0.1% PBS/ Triton‐X (Sigma, USA) was added and blocked for 20 min. An antibody (FSHR 1:500. FSTL3 1:500) was incubated with 8% Goat Serum in 0.1% PBS/ Triton‐X at room temperature for 2 hr, followed by washing with PBS three times. Then, the diluted second antibody (1:250) was incubated at room temperature for 1 hr in the dark, followed by washing in PBS three times. 15 μl ProLong (Life Technologies, USA) antifade reagent was added and Hoechst mixture (Life Technologies, USA) was carefully added on the fixed‐cell surface and cover glass slide; the samples were kept in the dark at 4°C until further observation.

### 2.3 Total RNA isolation and cDNA preparation

RNA of the tissues and GCs were extracted with TRIzol solution (Invitrogen, Thermo Fisher Scientific Inc., Waltham, MA, USA) and DNAse and adsorption columns (RNAprep pure Micro Kit DP420, Tiangen Biotech., Beijing, China) were used to remove possible DNA contaminations. The quality and concentration of extracted RNA were examined by 1% agarose gel electrophoresis and NanoDrop 2000 (Thermo Fisher Scientific Inc.). cDNA was synthesized using the One Step PrimeScript^®^ RT Reagent Kit (RR037A, Takara, Dalian, China).

### 2.4 RT‐qPCR

The expression levels of FSTL3 in ovaries during different reproductive stages and in follicles were measured via RT‐qPCR; β‐actin was used as internal control. Primers were designed by Primer 5.0, and sequences are shown in Table 1.

**TABLE 1:** Information of primer sequences for RT‐qPCR

Gene	Gene ID	Primer sequence (5'to3')	Length (bp)
*FSTL3*	XM_004009466	Forward: TCCAATTTCACCCACCCG	84
		Reverse: CCGCACTCCACGCCCTCG	
*β‐actin*	XM_004009466	Forward: CCAACCGTGAGAAGATGACC	97
		Reverse: CCAGAGGCGTACAGGGACAG	

Each 20 μl PCR reaction system contained 10 μl SYBR^®^ Premix Ex Taq Ⅱ (Tli RNaseH Plus), 2 μl cDNA, 0.8 μl forward primer, 0.8 μl reverse primer and 6.4 μl double‐distilled (dd) H_2_O. The qRT‐PCR was performed in the Roche LightCycler^®^ 480 II RT‐PCR system (Roche Applied Science, Branford, CT, USA). The procedure was 95°C for 15 min and then for 40 cycles at 95°C for 5 s, and 60°C for 30 s. All reactions were performed in triplicate. Non‐template reactions (replacing cDNA with RNase‐free H_2_O) were used as negative controls.

### 2.5 Western blotting

Protein extracted from complete homogenization of cells in immunoprecipitation buffer (Beyotime, Shanghai, China) was conducted according to the manufacturer's instructions. Protein was separated via SDS–PAGE, and then electrophoretically transferred to polyvinylidene fluoride (PVDF) membranes (MDBio Inc., Shandong, China).

Separated protein blots were incubated at 4°C overnight with 10 ml 5% skim milk powder in Tris‐buffered saline with Tween‐20 (TBST). An anti‐FSTL3 protein antibody (Abcam, Cambridge, UK, 1:2000) was incubated with PVDF membranes at room temperature for 1 hr, followed by washing with TBST three times, 10 min each. Then, diluted Goat anti‐Rabbit IgG (horseradish peroxidase labelled, HRP, Abcam, UK, 1:5,000) was incubated at room temperature for 1 hr, followed by washing in TBST three times, 10 min each. Stock solutions of 20 × LumiGLO and 20 × hydrogen peroxide were diluted to 1 × with ddH_2_O, which was added dropwise to the PVDF membranes and incubated in the dark for 1 min, taking images via the Molecular Imager^®^ Gel Doc™ XR System (BIO‐RAD, USA) in 30 min.

### 2.6 RNA Silencing

FSTL3 siRNAs were synthesized by Guangzhou Ribobio Co., Ltd., (Guanzhou, China) and the sequences are shown in Table 2. All reactions were performed in triplicate. The GCs were seeded in 6‐well plates at a density of 1 × 10^6^ cells/well in the medium of non‐antibiotic DMEM and incubated at 37°C in 5% CO_2_ in humidified air. Cells were transfected when they reached 70%–90% confluency. FSTL3 siRNA and Lipo3000 were diluted using Opti‐MEM following the manufacturer's instructions, and then mixed and added to cultured cells. Six hours after incubation, the medium was replaced with fresh serum‐free DMEM for 2–4 days extra. Then, cells were collected for RNA and protein preparation, and the supernatant was collected for E2 and P4 detection. Three targeting siRNAs were chosen to test the siRNA efficiency. Non‐targeting control siRNA was designed to prevent non‐specificity, and a blank control was used as transfection control.

**TABLE 2:** Primer sequences for *FSTL3* siRNA

siRNA ID	Target sequence	siRNA sequence
siRNA−1	ACACCGCCTGGTCCAATTT	Sence strand: 5’ ACACCGCCUGGUCCAAUUU dTdT 3’
		Antisence strand: 3’ dTdT UGUGGCGGACCAGGUUAAA 5’
siRNA−2	GTCTGAAGCGCTCTTTACT	Sence strand: 5’ GUCUGAAGCGCUCUUUACU dTdT 3’
		Antisence strand: 3’ dTdT CAGACUUCGCGAGAAAUGA 5’
siRNA−3	GCAACAACAACGTCACCTA	Sence strand: 5’ GCAACAACAACGUCACCUA dTdT 3’
		Antisence strand: 3’ dTdT CGUUGUUGUUGCAGUGGAU 5’

### 2.7 Statistical analysis

Duncan's multiple range test was used to calculate standard errors among replicated samples using IBM SPSS Statistics (v. 24.0; IBM Corp., Armonk, NY, USA). The 2^−ΔΔCT^ method was used to calculate relative mRNA levels (Livak & Schmittgen, 2001). Analysis of variance (ANOVA) was used to examine significant differences of expression levels between samples using SAS v. 9.2 (SAS Institute Inc., Cary, NC, USA).

## 3 RESULTS

### 3.1 Dynamic changes of ovine hormone levels during different reproductive stages

**FIGURE 1:**
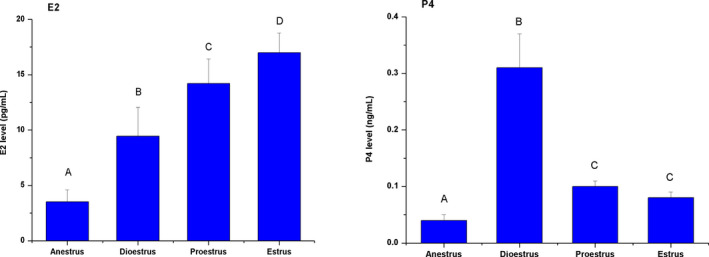
Serum concentrations of E2 and P4 at different reproductive stages. Concentrations of E2 and P4 were measured via radioimmunoassay. E2: oestrogen; P4: progesterone. Different capital letters indicate significant difference among the stages (*p* < .01)

As shown in Figure 1, the levels of E2 and P4 in anestrus were significantly lower compared to those during the stages of oestrus (*p* < .01). During the stages of oestrous cycle, the levels of E2 in both proestrus and oestrus were higher than in the dioestrus (*p* < .01). Furthermore, the level in the oestrus was higher than that in the proestrus (*p* < .05). With regard to P4, the level in the dioestrus was higher than in both proestrus and oestrus (*p* < .01). These results showed that the concentration of E2 gradually increased from the anestrus stage to dioestrus, proestrus and oestrus stages, indicating that their high levels can promote the oestrus while low levels inhibits the oestrus. P4 level was highest during the luteal phase when the corpus luteum showed at its maximum size.

### 3.2 Gene expression profiles of sheep FSTL3 mRNA

**FIGURE 2:**
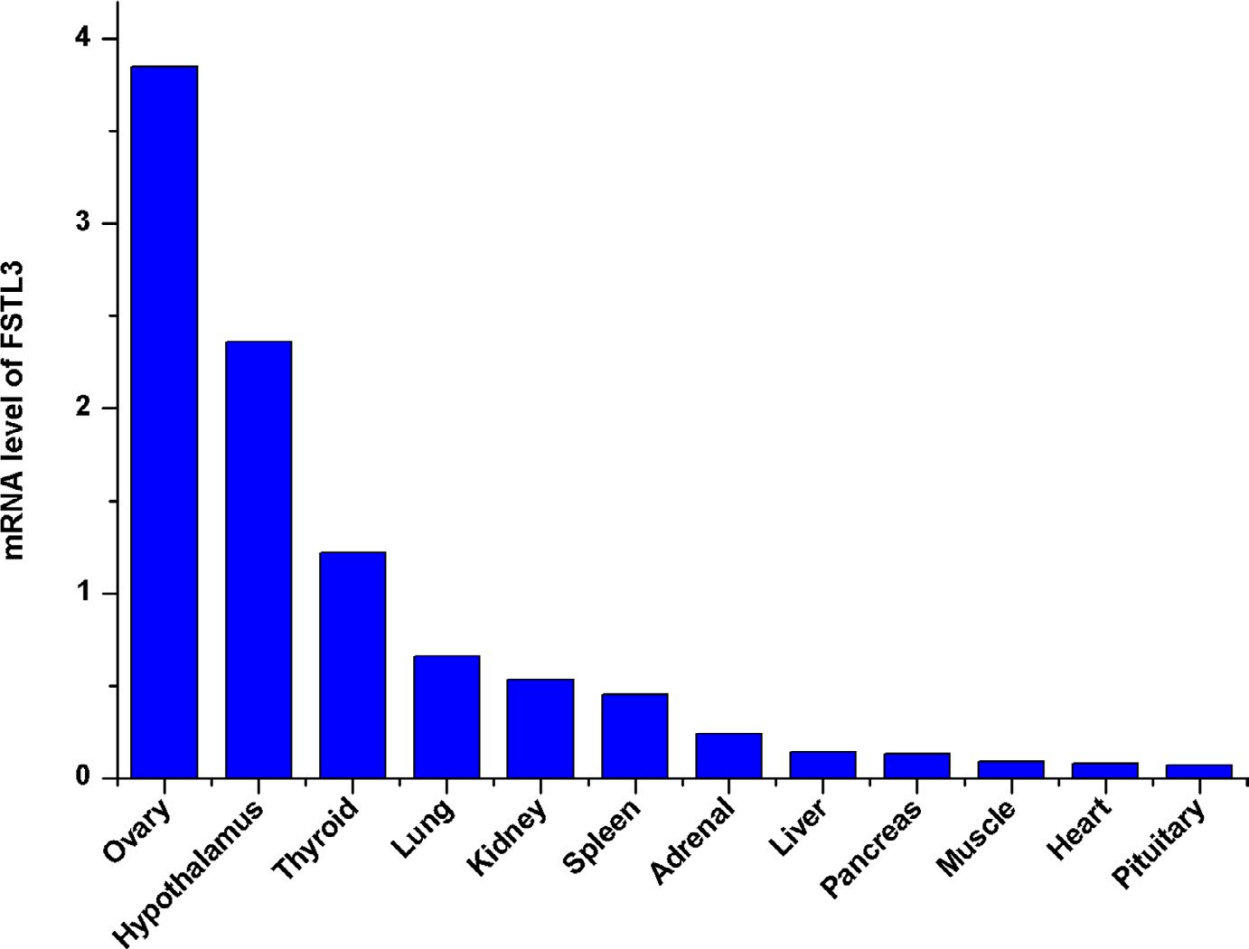
mRNA expression level of the FSTL3 gene in different tissues. The mRNA distribution of FSTL3 in different tissues was detected via RT‐qPCR

FSTL3 mRNA expression levels were detected in different tissues. RNA was extracted from the tissue in different reproductive stages. As shown in Figure 2, expression levels of FSTL3 were highest in the ovary, hypothalamus and thyroid. Additionally, they had low expression levels in other tissues, including muscle, kidney and lung. Our results indicate that FSTL3 may play a role in reproductive process.

### 3.3 Expression pattern of the FSTL3 gene in the ovine ovary during different reproductive stages

**FIGURE 3:**
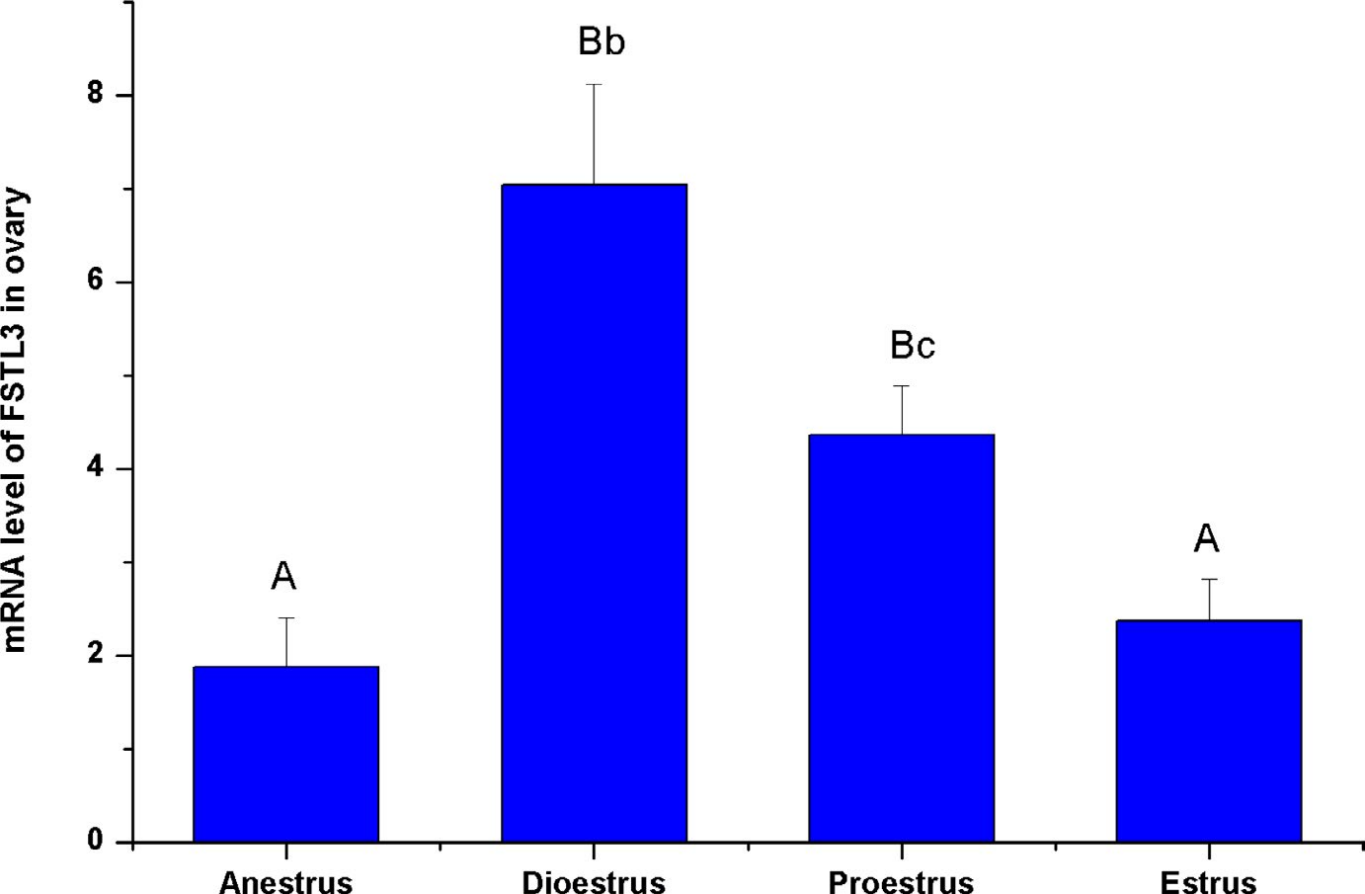
mRNA profile of FSTL3 in sheep ovary during the different reproductive stages. The mRNA profile of FSTL3 during the stages of anestrus, dioestrus, proestrus and oestrus was measured via RT‐qPCR. The different capital letters indicate significant differences among the stages at the *p* < .01 level, and different lowercase letters indicate significant differences among the stages at the *p* < .05 level

As shown in Figure 3, the FSTL3 expression level in the ovary was significantly higher during the dioestrus compare to the other three stages. The lowest levels can be found at oestrus and anestrus stages, which were significantly lower than levels at dioestrus and proestrus stages (*p* < .01); the expression levels between oestrus and anestrus have no significant difference.

### 3.4 Interference of FSTL3 in ovine ovary GCs cells

**FIGURE 4:**
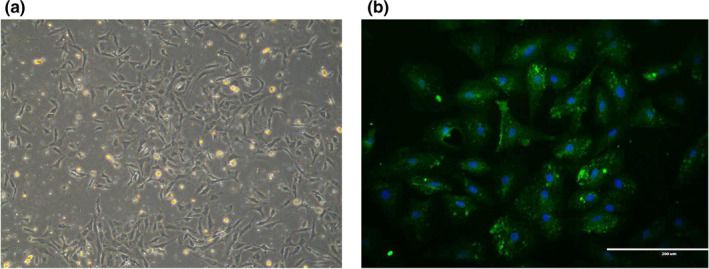
Ovarian granulosa cells of sheep (a, b). (a) Images of sheep granulosa cells after cultivation for 24 hr. (b) FSHR expression in sheep granulose cells. Green indicates the cytoplasm, blue the nucleus

**FIGURE 5:**
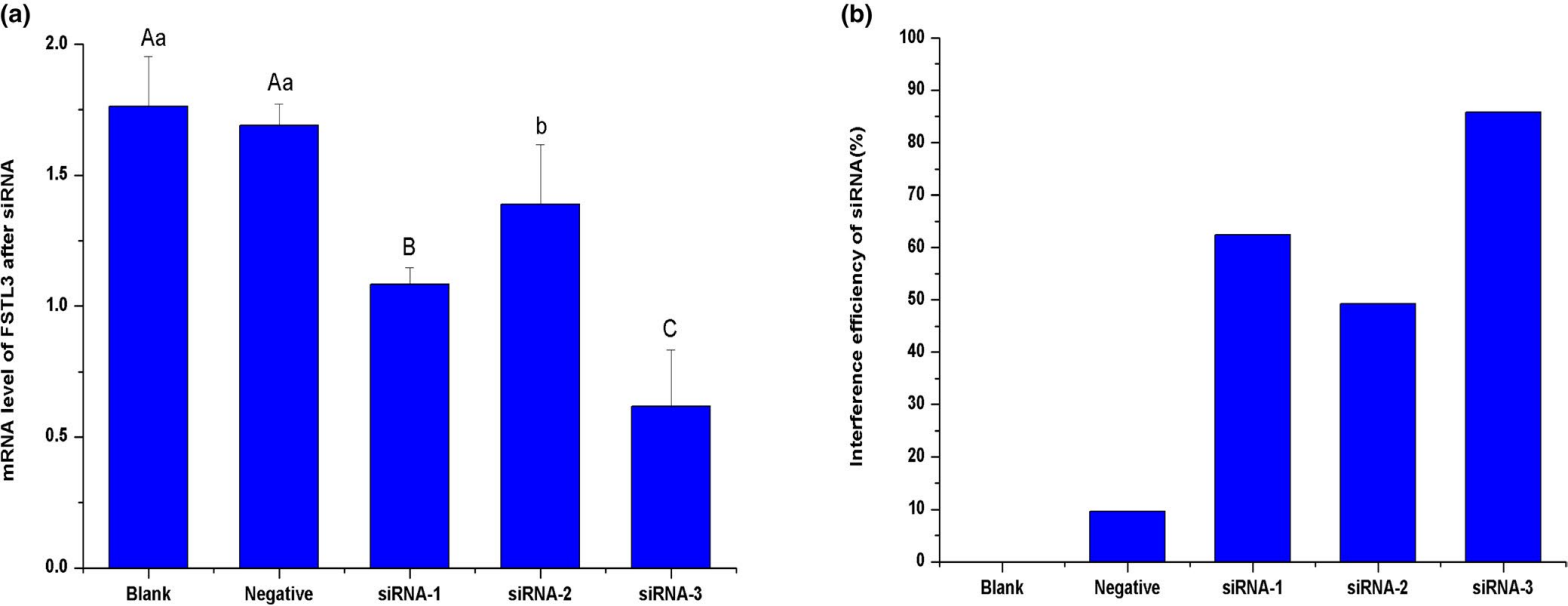
mRNA expression of FSTL3 gene after transfection and interference efficiency of siRNA (a, b). (a) The mRNA expression level of FSTL3 gene was detected via RT‐qPCR after transfection with siRNA‐1, siRNA‐2 and siRNA‐3. (b) The interference efficiencies of siRNA‐1, siRNA‐2 and siRNA‐3 were analysed according to a blank control. Blank: blank control; Negative: negative control

**FIGURE 6:**
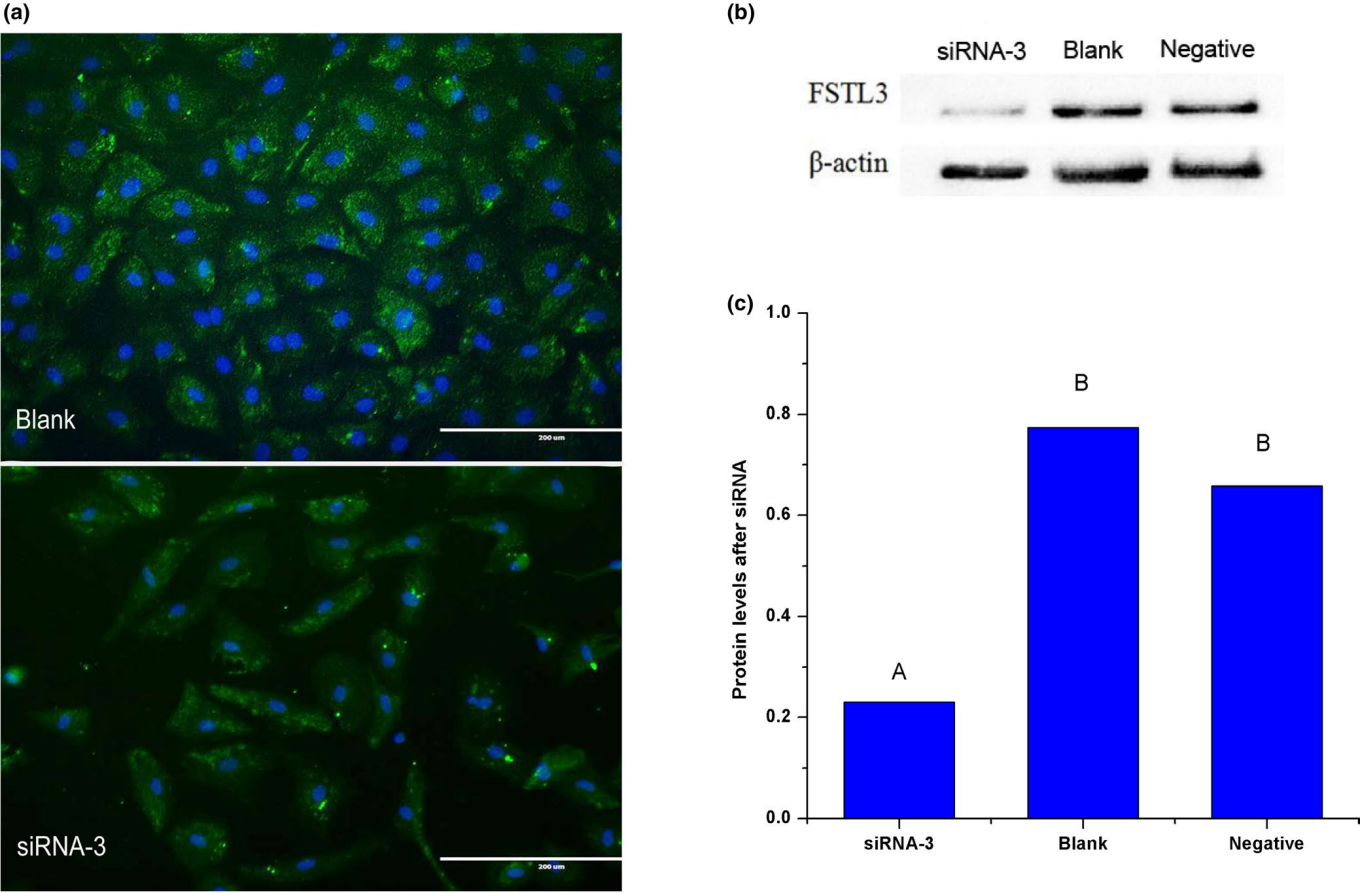
Changes of FSTL3 protein in granulosa cell after transfection with SiRNA‐3 (a, b, c). (a) Detection of FSTL3 inhibition in granulosa cell via immunofluorescent staining. Blank: blank control; Green indicates the cytoplasm, blue indicates the nucleus. (b) and (c) show the detection of the inhibition of FSTL3 in granulosa cell via Western blotting. Different capital letters indicate significant differences among the stages (*p* < .01)

GCs derived from sheep ovaries were spindle‐shaped cells and grew as an adherent cell monolayer (Figure 4a). As shown in Figure 4b, the purity of primary GCs was very high, with >90% of GCs expressing the follicle‐stimulating hormone receptor (FSHR) at 48 hr after isolation, as shown via immunofluorescence staining. To identify the function of FSTL3 in sheep GCs, three pairs of target‐specific siRNAs (siRNA‐1, siRNA‐2 and siRNA‐3) were designed. GCs were transfected with these siRNAs, and FSTL3 mRNA expression was detected after 48h transfection in the interference group, blank group and negative group, respectively. As shown in Figure 5, siRNA‐3 was highly effective in knocking down the target gene while the interference efficiency was up to 85.84%. After siRNA‐3 transfection, FSTL3 protein in GCs was significantly inhibited in interference group compared to control groups (*p < *.01) as shown in Figure 6 by immunofluorescence and Western blotting.

### 3.5 Changes of E2 and P4 levels in GCs after FSTL3 of RNAi

**FIGURE 7:**
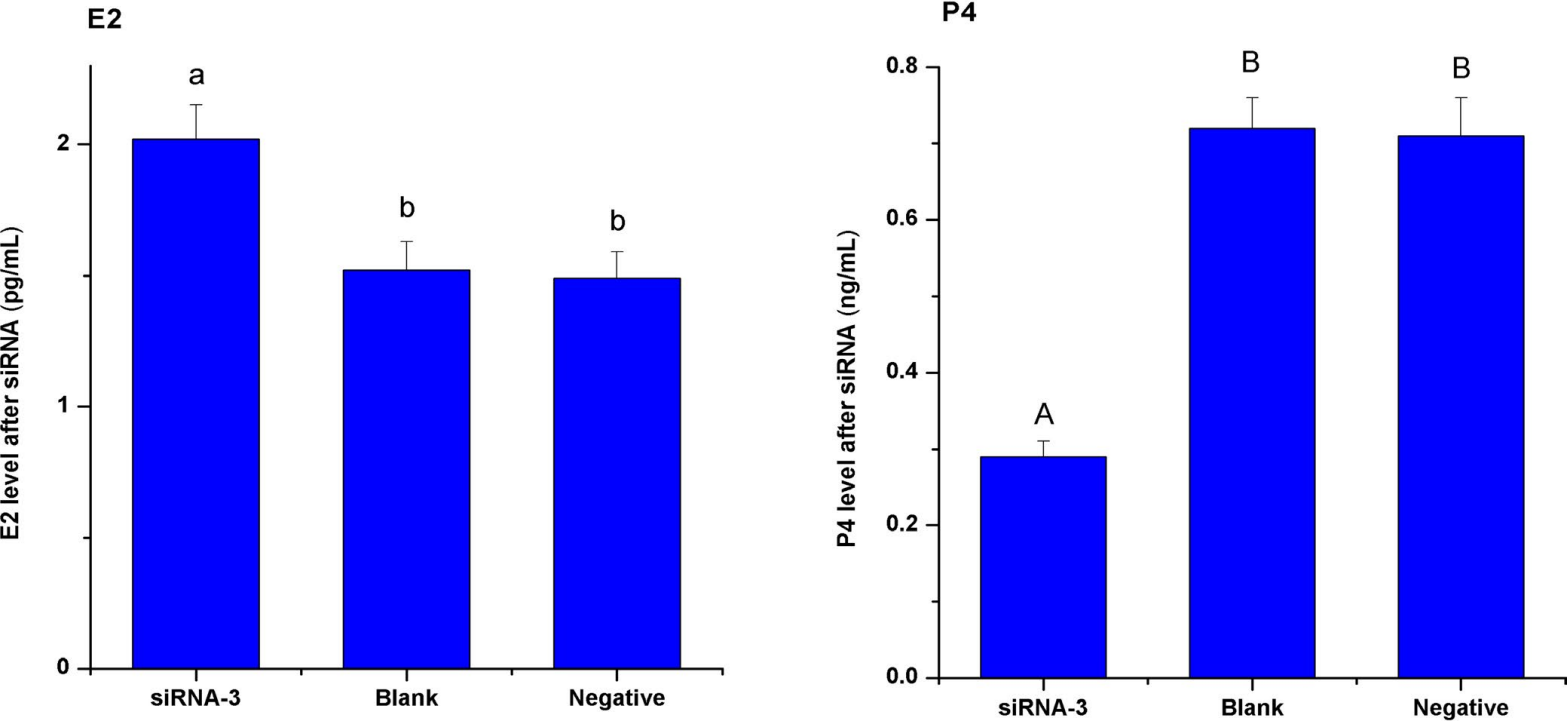
Levels of E2 and P4 in granulosa cell after transfection with siRNA‐3. The concentrations of E2 and P4 were measured via radioimmunoassay. E2: oestrogen; P4: progesterone. Different capital letters indicate significant differences among the stages (*p* < .01), different lowercase letters indicate significant differences among the stages (*p* < .05)

As shown in Figure 7, after siRNA‐3 transfection for 48 hr, E2 levels in GCs were 1.52 ± 0.11 pg/ml, 1.49 ± 0.10 pg/ml and 2.02 ± 0.13 pg/ml in blank, negative and interference group, respectively measured by radioimmunoassay. Obviously, the E2 level was significantly higher in interference group than in both the blank and negative groups (*p < *.05, while the trend of P4 level changing was opposite to E2 (*p < *.01). Furthermore, the levels were 0.72 ± 0.04 ng/ml, 0.71 ± 0.05 ng/ml, and 0.29 ± 0.02 ng/ml in blank, negative and interference group, respectively.

## 4 DISCUSSION

Seasonality of mammals was caused by hypothalamic gonadotropin‐releasing hormone (GnRH) secretion, and it has been confirmed that the transition between breeding and non‐breeding seasons primarily produces a drastic reduction of the LH pulse frequency. A complete oestrous cycle can be divided into a follicular and a luteal phase. Ovarian steroids—mainly estradiol (E2)—can carry out negative feedback actions following GnRH secretion, and the oestrus receptor alpha (ERa) is the predominant mediator of this feedback effect during seasonal breeding (Banerjee et al., 2016; Matsuda et al., 2012).

Our results showed that E2 and P4 levels had significant changes during the transition from dioestrus to oestrous stages. It has been reported that a big corpus luteum can be found in the ovary at the dioestrus stage which will secrete P4. At the oestrous stage, a large follicle was found instead of the corpus luteum secreting high E2 concentration (Beard & Lamming, 1994; Cunningham et al., 1975; Sogorescu et al., 2012; Thorburn et al., 1969). Our results indicated that AFW sheep were used displayed same hormone secretion pattern as other seasonal breeding sheep breeds.

In the present study, expression distribution in different tissues of ovine FSTL3 was demonstrated. The tissue expression of ovine FSTL3 mRNA in sheep was similar as that in mice, rats and humans. FSTL3 mRNA expression in reproductive tissues was higher than in other tissues (Roberts, 1997; Tortoriello et al., 2001; Wijayarathna et al., 2018; Winnall et al., 2013). The higher expression level of FSTL3 in the ovary would indicate that FSTL3 may participate the reproductive regulation in ovary (Hedger et al., 2011; Wijayarathna et al., 2018). From our results, FSTL3 expression level was significantly elevated during the dioestrus compare to the other three stages. The lowest levels were found during oestrous and anestrus, which is consistent with previous RNA‐sequencing data (Di et al., 2014). The results of the FSTL3 expression level, hormone concentration and reproductive appearance suggest that the FSTL3 gene in the ovary may have a relationship with E2 and P4 concentration, and control the initiation and maintenance of the oestrous status in sheep.

Functional analysis of FSTL3 was conducted in primary granulosa cells of sheep. Results showed that the concentration of E2 secreted by GCs was significantly increased after FSTL3 inhibition, while the P4 concentration was significantly decreased. Since GCs are the main functional cells in the ovary, exerting a very important role in regulating the physiological function of the ovary, oocyte quality and embryo development. The proliferation and apoptosis of GCs are affected by hormones, cytokines and genes. Both E2 and P4 can affect the apoptosis of GCs (Billig et al., 1993; Wu et al., 1998). This may indicate that FSTL3 can inhibit the concentration of E2 and promote the concentration of P4 secreted by GCs. Previous studies showed that E2 and P4 can improve oocyte quality, inhibit granulocytes apoptosis and resist follicular atresia (Billig et al., 1993; Makrigiannakis et al., 2000; Peluso & Pappalardo, 1994; Wu et al., 1998). Therefore, this suggests that FSTL3 may influence the sheep oestrous status by regulating cell proliferation and apoptosis of ovary GCs. However, sheep oestrus is under the sophisticated network of regulatory signals, which includes light, hormone levels, nutrition and genetic factors. Since the FSTL3 gene is a member of the TGF‐β pathway, many genes as SMAD3, SMAD4, TGF‐α and TGF‐β can affect the transcription of FSTL3 (Ciarmela et al., 2004; Maguer‐Satta et al., 2001, 2006). FSTL3 being a mouse follistatin‐like protein contains two follistatin domains. It can act binding activity for both activin and BMP‐2 like follistatin protein as previously reported (Tsuchida et al., 2000). In the sheep ovary, granulosa cells are the main type of cell in the ovary responsible for producing and secreting follistatin in most species. In our study, the concentration of E2 was inhibited while the concentration of secreted P4 was promoted after interference of FSTL3 expression in GCs. It means that FSTL3 can participate in folliculogenesis of oocyte. In addition, the concentration of follistatin production varies according to the extent of differentiation of granulosa cells. It was shown in our data that expression level of FSTL3 in the sheep ovary changes with the reproductive status which are corresponding to different extents of differentiated granulosa cells, and FSH and GnRH may mediate follistatin protein also including FSTL3 production in granulosa cells which can lead to alteration of E2 and P4 concentrations. We suggested a hypothesis that FSTL3 expression can positively regulate the hormone secretion according to the extent of differentiation of granulosa cells corresponding to different reproductive status, and it may give the opportunity to alter the oestrous cycle of sheep by manipulating the FSTL3 expression.

## 5 CONCLUSIONS

In this study, we examined the relationship of FSTL3 expression and the reproductive status during sheep oestrous cycle. It was preliminarily shown that FSTL3 can affect E2 and P4 concentrations by switching its own expression level corresponding to different reproductive status. The precise mechanism involved in how FSTL3 can bind to activin or TGF‐β family members and then mediate concentration alteration of reproductive hormones during seasonal change should be identified in the future.

## CONFLICT OF INTEREST

None of the authors have any conflict of interest to declare.

## AUTHOR CONTRIBUTIONS

N.L. and M.C. conceptualized and designed the project; J.H. and S.Y. collected the samples; Q.L., M.L. and Z.G. analysed the data; J.H. and Q.L. wrote the manuscript; N.L. revised the manuscript; J.L., R.D., W.H. and X.W. participated in data analysis and figure preparation. All authors approved the submitted version for publication.

## Data Availability

Data sharing is not applicable to this article as no new data were created or analysed in this study.
